# Barriers and facilitators to physical activity for young adult women: a systematic review and thematic synthesis of qualitative literature

**DOI:** 10.1186/s12966-023-01411-7

**Published:** 2023-02-27

**Authors:** Bo Peng, Johan Y. Y. Ng, Amy S. Ha

**Affiliations:** 1 Department of Sports Science and Physical Education, The Chinese University of Hong Kong, Hong Kong, China; 2 Faculty of Education, The Chinese University of Hong Kong, Hong Kong, China

**Keywords:** Young women, Physical activity, Barriers, Facilitators, Social-ecological model, Qualitative synthesis

## Abstract

**Background:**

Physical activity (PA) has many benefits in preventing diseases and maintaining physical and mental health. Women, in particular, can benefit from regular PA. However, women’s PA did not increase over the past decade globally, and the situations faced by women are often gender-specific. Healthy young adult women's PA does not receive as much attention as older women and adolescent girls, yet, they face the same situation of low level of PA. This review aims to explore and synthesise the self-identified barriers and facilitators to young adult women's participation in PA from qualitative research studies and offer suggestions for future studies and programs designed for this population.

**Methods:**

A systematic search was conducted in Pubmed, Web of Science, Scopus, Medline, and SPORTDiscus for studies published between January 2000 to February 2022 to identify qualitative studies on the barriers and facilitators of young adult women’s PA between ages 18 to 40. The search yielded 694 studies initially, of which 23 were included. The research quality of included studies was appraised using the Critical Appraisal Skills Programme (CASP) tool. Data were extracted and thematically analysed based on the tenets of the social-ecological model (SEM).

**Results:**

Identified barriers and facilitators were grouped into different levels of the SEM, with the most frequently cited factors being time, body image and societal beauty standards, family duty and social support, religious and cultural norms, organisation and community facilities and environment, safety issues and physical environment. Descriptive data were thematically analysed and synthesised in line with the five levels: body image, health and beauty; multiple roles, support, and PA; religious identity, cultural identity, and PA; safety issues and women’s fears.

**Conclusions:**

This qualitative synthesis revealed in-depth information on barriers and facilitators influencing young adult women’s PA. It highlighted that the factors young adult women face are diverse at different levels yet holistic and intertwined. Future studies on young adult women’s PA should address the social-cultural influence and would benefit from applying multilevel strategies employing the SEM model. It is critical to create an open and inclusive environment and offer more opportunities for women.

**Trial registration:**

PROSPERO CRD42021290519.

**Supplementary Information:**

The online version contains supplementary material available at 10.1186/s12966-023-01411-7.

## Introduction

It is well-documented that women of all ages benefit from regular physical activity (PA) [1] in preventing diseases and maintaining physical and mental health [2–6]. However, physical inactivity is already a pandemic with far-reaching health, economic, environmental and social consequences [7]. Women were nearly 8% less active than men worldwide and remained at a lower level of PA in the past decades [8]. A global study across 142 countries concluded that without any change in men’s PA, only a slight increase in women’s PA [9] would achieve the WHO global target of reducing physical inactivity by 10% by 2025 [10]. Thus, there is a need to tackle the gender gap [11] and improve women’s PA levels.

Despite research showing that men are biologically more active than women [12], women’s PA may also be negatively influenced by societal expectations and cultural norms. These social-cultural expectations usually become more pronounced and significantly impact young adulthood, about 18 to 40 years [13, 14]. Stepping into young adulthood means women are moving into a pivotal point of life transitions characterised by multiple role adaptations and a wide diversity of lifestyles [15]. During this time, many environmental (e.g., from home to university or work), social (e.g., marriage), and personal life events (e.g., motherhood) [16, 17] may occur and, consequently, could have both detrimental and beneficial impacts on women’s well-being and PA [18–20]. However, women generally face a sharp decline in PA from the transition to young adulthood [16]. Especially women of today are expected to fulfil various roles, such as being caregivers, mothers, wives, and dedicated employees; while role expectations rise, the level of PA falls even more [20–22].

Compared to other groups of PA, such as children, adolescents, the elderly, and the population with disease, healthy young adults are sometimes overlooked [23]. Recent studies found a rising trend in chronic diseases in young adults that usually affect middle-aged and older people [24]. Further, studies found a strong link between maintaining healthy lifestyles throughout young adulthood and lower cardiovascular disease risk in middle age [25, 26]. This suggests that physically active young adult women may become healthy middle-aged to older women and further reduce the burden on healthcare systems. Some existing reviews have presented factors influencing women’s PA at different ages and situations [27–31], but most adopted a quantitative approach. However, the results of these studies were often reported and interpreted without contextual considerations. Without an embedded context, it is difficult to comprehend the reasons and underlying connections between these factors and generate practical implementations. Hence, using the qualitative synthesis technique, this review was grounded in young adult women's perceptions, experiences and narratives and aimed at interpretative rather than only aggregative [32]. Gathering data and findings from qualitative studies on the same topic across various contexts can present a broad review of young adult women’s behaviours, emotions, and attitudes towards PA [33].

Research on correlates or determinants of PA has burgeoned and primarily concentrated on individual-level characteristics in high-income countries, namely age, gender, health status, lifestyle, self-efficacy, and motivation [34]. However, this could sometimes fall into a victim-blaming ideology [35, 36] that dismisses the inevitable subliminal influence of the broader context. Hence, this review employed the social-ecological model (SEM) [36] as the underlying framework. Many studies and reviews also used this model to layer factors that might have influenced individual behaviours. The SEM acknowledges the impact of human behaviour and development beyond the individual level and divides the influences into five levels: intrapersonal, interpersonal, organisational, and environmental or policy levels to present visual depictions of the dynamic relationships between each level [37]. In health research and practices, the SEM takes a comprehensive view [36], paying particular attention to influences outside the health sector [34], such as the social-cultural and physical environment, which is in line with the socio-cultural impact young adult women's may face with their PA that this review aimed to explore.

Based on the above, this study aimed to provide a systematic review of qualitative studies exploring the barrier and facilitators of young adult women’s PA at different levels of SEM for better generalisation and targeting efficiency of PA strategies. The aims were four-fold: 1. describe the characteristics and methodologies of qualitative studies conducted on this topic; 2. identify barriers and facilitators for young adult women’s PA and categorise them into corresponding levels within the SEM; 3. synthesise the narratives from qualitative studies and present themes based on tenets of the SEM; 4. Identify significant barriers and facilitators and their connections to young adult women’s PA.

## Method

The review was conducted following the Preferred Reporting Items for Systematic Reviews and Meta-Analyses (PRISMA) guidelines [38] and the Enhancing Transparency in Reporting the Synthesis of Qualitative Research (ENTREQ) guidelines [33]. A protocol for this review was published in PROSPERO (registration number: CRD42021290519).

### Search strategy

A systematic search of Medline, Pubmed, Scopus, SPORTDiscus and Web of Science from January 2000 to February 2022 was performed to identify studies that met the inclusion criteria. The search terms included keywords and MeSH headings using Boolean/Phrase on a combination of keywords specifying the physical activity, barriers, facilitators, and young adult women yielded 694 studies. All searches used subject headings where available (see Additional file 1 for an example of search strategy). Citation tracking was also conducted through Google Scholar and Web of Science. The first author also screened the reference lists of included manuscripts to identify other studies that may have met our inclusion criteria.

The first author conducted the initial search from databases and screened the titles and abstracts of the search results; any ambiguity was discussed with the second author. Studies shown as conference abstracts were manually searched for full text on the Web of Science. The first and second authors separately screened the studies that advanced to the full-text screening stage.

### Inclusion criteria

The inclusion criteria are displayed in Table 1.

**Table 1:** Search strategy

Inclusion criteria
**Participants**
Young adult women aged between 18 to 40
Participants with healthy physical and mental status
**Design**
Qualitative studies with descriptive data
Full-text articles published in English
**Outcome measures**
Participant's attitudes or perceptions of PA
Participants perceived barriers or facilitators to PA
**Clarification**
** PA:** Including leisure-time physical activities and structured exercise, sports participation
** Healthy:** Participants without outstanding physical or mental disorders/disabilities. Studies on the population in recovery/rehabilitation/potential medical risk were excluded
** Pregnancy:** Studies on pregnancy, the prenatal period, and the postpartum period were excluded because they pertain to a particular biological stage of women where the barriers and facilitators are strongly tied to pregnancy and lack the applicability of the age of young adult women in this review

### Selection process

**Fig. 1:**
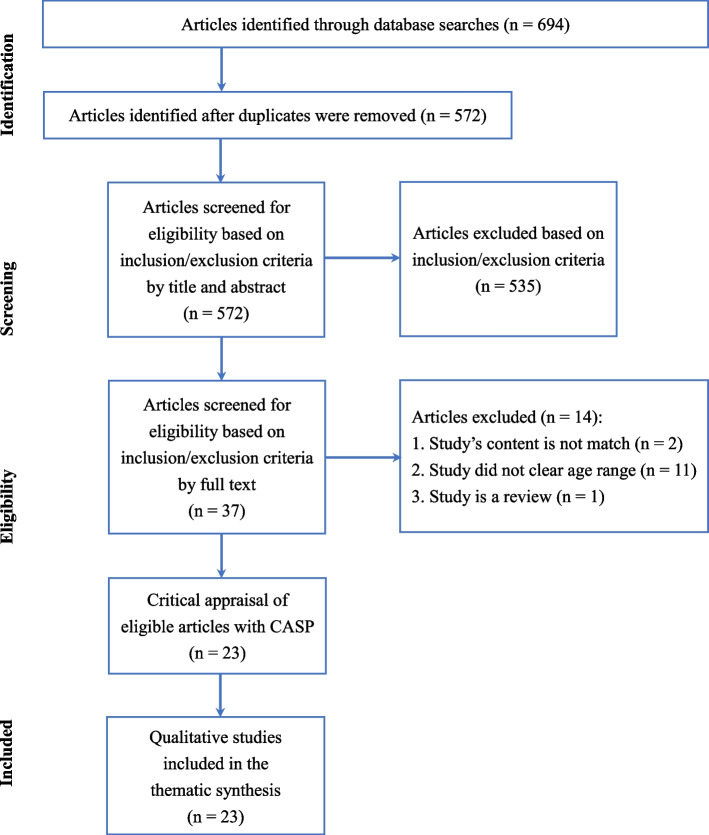
PRISMA flow diagram

After removing duplicates from the initial 694 studies, hierarchical screening was performed by the first and second authors, respectively, following the PRISMA guidelines [38] (see Fig. 1). Studies that met the following exclusion criteria were removed sequentially in the following order: 1. studies without full-text or not published in English; 2. studies of non-healthy women or not within the 18 to 40 age range (such as disease, rehabilitation, adolescents, old, etc.); 3. studies used quantitative method; 4. studies meet the inclusion criteria yet focused on pregnancy, prenatal and postpartum period of women. Thirty-seven qualitative studies were screened full-text by authors for eligibility. Fourteen studies were excluded because there were no age characteristics (18 to 40 years) in the quotes and results. Two were excluded because they focused on special populations (one with a disease, one with pregnancy-related), and one study was excluded as a review. A total of 23 studies were included for critical appraisal.

### Quality appraisal

The final 23 studies included were appraised using the Critical Appraisal Skills Programme (CASP) qualitative research quality tool (see Additional file 2 for Critical appraisals by the CASP) [39], which is a widely used tool for quality appraisal in health-related qualitative evidence syntheses, with endorsement from the Cochrane Qualitative and Implementation Methods Group [40]. The first and second authors independently conducted the assessment. Any discrepancies were discussed and reached a consensus between all authors. Each study's research design was appropriately justified. Five studies did not state the ethical concerns or whether they used quotes ethically to support their findings. Nine studies failed to adequately disclose the researcher's or interviewer's roles and the potential for bias in the results. Two studies did not display sufficient and vigorous data or provide thick descriptions to support their findings. All the findings were tied to the study's original purpose, and all the studies discussed the findings' trustworthiness. All the twenty-three studies were regarded as valuable; none were excluded for methodological concerns.

### Data extraction and analysis

Extracted data from each study include 1. author and year of publication, 2. country, 3. sample characteristics, 4. qualitative data collection methods, 5. type of analysis, and 6. study results or findings (see Additional file 3 for Characteristics of the included qualitative studies). Each study's results were regarded as text in the titled "results," "findings," sections or similar parts. Fort studies’ results not only focused on young adult women (i.e. age from 18 – 60 years) or PA (i.e. health behaviour), data were retrieved including 1. results that were identified as specific to women between the ages of 18 and 40; 2. results that claimed to apply to all participants; 3. results only pertinent to PA.

The first and second authors independently extracted data from studies and coded the information into Excel and Word templates developed for this study. No other software or services were used to manage the review processes.

### Synthesis methods

A combined inductive and deductive approach was applied in the analyses. Two authors independently coded the extracted data line-by-line, using thematic analysis inductively to categorise the data and develop new themes ‘beyond’ the reports from the original studies. A combination of direct-to-analysis and extraction techniques were used in the analysis to avoid preconceptions related to original documents abstracted from the context of articles in which they are embedded [41]. Afterwards, themes were deductively grouped into five different levels of the SEM. Themes from the original studies were then collected and grouped into the SEM for comparison to the newly identified themes. Eventually, the authors compared and combined the new and original themes and reached a consensus on the final result.

## Result

### Barriers and facilitators of young adult women’s PA

The identified barriers and facilitators of young adult women’s PA were shown in Table 2 and Table 3, as well as the contribution of each study.

**Table 2:** Facilitators of young adult women’s PA

SEM	Themes	Facilitators	Articles ID
**Intrapersonal**	Overall health	Maintain/improve health, prevent diseases	All mentioned
	Mental aspects	Feel good/energetic (instant & long term)	3, 4, 6, 7, 9, 10, 15, 17, 18, 19, 21
		Stress relief and relaxation	2, 4, 7, 8, 13, 17, 18, 21
	Physical aspects	Weight control (loss/gain/maintain)	1, 3, 4, 5, 6, 7, 11, 13, 14, 17, 18, 19, 20, 21, 23
		Appearance/body image enhancement	1, 3, 4, 5, 6, 7, 9, 14, 18, 20, 21, 22
		Learn/master a skill	5, 8
	Life enhancement	Healthy lifestyle and habits	3, 5, 6, 7, 8, 9, 13,18, 20, 21
		Personal time/down time	3, 5, 6, 7, 9, 13, 22, 23
	Attitudes	Positive attitude (interests, enjory, fun)	1, 4, 5, 6, 8, 11, 13, 16, 18, 19, 20, 21, 23
		Setting priorities (PA-prioritized)	2, 6, 13, 17, 22
	Competence	PA knowledge	4, 8, 14, 17, 18, 21
		PA skills	1, 5, 9, 15,
	Self awareness	Self-Esteem and confidence	5, 6, 9, 18,
		Self-efficacy and obligation	3, 6, 7, 13, 15, 17, 18
**Interpersonal**	Supports (attitude)	Support from spouse	2, 9, 10, 11, 14, 19, 22
		Support from parent	1, 3, 6, 12, 14, 18
		Support from/for children	8, 14, 22
		Support from friends	2, 3, 3, 5, 8, 10, 11, 13, 14, 15, 17, 18, 20, 21, 22
	Social-relatedness	Socialising (PA friends/Team sports)	2, 5, 6, 7, 8, 10, 11, 13, 15, 17, 18, 20, 21,
		Active social life	3, 10, 13, 18, 20, 21, 22, 23
	Experts/Authority	Role models/To be a role model	1, 3, 4, 5, 6, 7, 10, 17, 20
		Professional advice/feedback	2, 14, 18, 19, 21
		Leadership from instructors	1, 3, 7, 10, 11, 13, 19, 21
**Organizational**	Home/Family	Active family environment/traditions	1, 2, 3, 6, 14, 15, 21
		Substantial help from family members	3, 8, 13, 18, 22
	Community	Accessible facilities and places	1, 2, 6, 11, 16, 20, 21, 22
		Active community/neighborhood	2, 17, 20
	School	Positive experience in PE	16, 18, 22, 23
	Work agency	Physically active job	11, 13
	PA organization	Affordable price or free access	2, 4, 13, 18, 20, 21
		Accessibility (information, facilities)	2, 4, 5, 6, 11, 13, 18, 20, 21
		Flexible programs (schedule, level, content)	2, 3, 5, 13, 19, 21, 23
		Cultral sensitivity & inclusiveness	1, 3, 4, 6, 10, 14, 16, 20,
		Women-centered (by women for women)	1, 8, 13, 14, 16, 17
		Family-oriented (especially mother&child)	14, 17, 21
**Community**	Cultural norms	Societal standards of beauty/success	3, 6, 7, 18, 22
		Good/super mother identity	9, 13
		Sex segregation (women only)	1, 4, 6, 8, 10, 12, 16, 22, 23
		Flexible dress code	1, 4, 6, 12, 16, 21, 22
	Religious aspects	Acceptance of religious identity and minority	1, 4, 6, 16, 21
**Environmental**	Environment	Natural environment (park, fresh air, etc.)	6, 15, 17, 20, 21
		PA-friendly environment (walk & bike lane, etc.)	3, 4, 11, 17, 20, 21, 22
		Safety environmnet	10, 14, 21
	Transportation	For commuting	7, 8, 11, 13, 21
		Convinient transportation	19, 21

**Table 3:** Barriers of young adult women’s PA

**SEM**	**Themes**	**Bacilitators**	Articles
**Intrapersonal**	Lack of time		All
	Attitudes	Health is not controllable	10, 13
		PA as a sign of modernity and wealth	8, 14
		Lack of interests (dislike for PA)	1, 2, 3, 4, 7, 10
		Competing priorities (others people/other things)	4, 6, 7, 13, 15, 17, 20
	Competence	Lack of PA knowledge/awareness	4, 5, 8, 10, 16, 17, 18, 20, 21
		Lack of PA skills	1, 5, 20
	Self awareness	Lack of motivations	4, 7, 8, 10, 11, 15, 20, 23
		Lazy or tiredness	1, 4, 7, 9, 10, 11, 13, 17, 23
		Lack of self-efficacy (hard to maintain)	2, 7, 20
**Interpersonal**	Commitments	Family chores & caring duties	2, 3, 6, 8, 9, 10, 11, 13, 14, 16, 17, 19, 22, 23
		Feeling guilty about doing PA for herself	9, 23
	Embarrassment	Fear of judgement of body & looks	4, 18, 19, 20, 22
		Fear of judgement of skills & knowledge	3, 17
	Social relatedness	Lack of social support/relatedness	4, 5, 10
	Experts/Authority	Lack of good instructors	1, 4, 5
**Orgnizational**	Home/Family	Inactive family environment/traditions	1, 10, 12, 14, 20, 21, 23
		Lack of substantial help from family	1, 2, 3, 8, 9, 10, 11, 12, 13, 14, 16, 18, 20, 22, 23
	Community	Inactive community/neighborhood	6, 11, 17, 18, 20
	School	Negative PE experience	1, 4, 12, 16, 18,
		Academic priority	1, 14, 18, 20, 21, 22
	Work agency	Excessive work loads	9, 11, 17, 19
	PA organization	Financial issue (unaffordable)	2, 4, 11, 13, 17, 19, 20, 21
		Lack of accessbility (information, facilities)	2, 3, 4, 6, 8, 13, 17, 18, 21, 23
		Lack of tailered programs	1, 2, 3, 5, 8, 13, 16, 17, 19
**Community**	Cultural norms	Pressure of mainstream/social media beauty	3, 18, 22
		Lack of PA culture	1, 4, 6, 10, 12, 14, 21, 22, 23
		Patriarchal family setting	6, 8, 9, 11, 13, 14, 23
	Gender norms	Meeting traditional role duties (mother/wife identity)	6, 9, 11, 13, 14, 17, 22, 23
		Gender norms limit (unfeminine)	1, 3, 4, 6, 8, 12, 13, 14, 16, 20, 21, 22, 23
		Men/others gazing	4, 6, 10, 14, 19
	Religious aspects	Religion’s precedence over PA	1, 4, 8, 10, 12, 32, 23
		Public modesty (co-ed environment, dress code)	1, 4, 6, 8, 10, 11, 12, 14, 16, 21, 22, 23
		Insufficient understanding from others/community	1, 6, 11, 12, 14, 16
**Environmental**	Weather conditions		2, 4, 10, 16, 17, 21
	Physical environment	Lack of space at home	13
		Lack of “proper” places (park, gym, etc.)	4, 6, 11, 17
	Transportation	Sedentary society	8, 10, 11, 13, 17, 23
		Inconvenience transportation	2, 3, 4, 13, 17, 19, 21
	Safety issues	Unsafe environment	8, 10, 11, 20, 21, 22
		Potential risks for women	8, 11, 20, 21, 22

### Qualitative synthesis

#### Intrapersonal & interpersonal & community level: body image, health and beauty

##### Body dissatisfaction & health

Health is the fundamental and most common intrapersonal facilitator for everyone's PA participation. However, it may not be the most popular or motivating factor for young adult women. Previous findings from epidemiological studies consistently showed that many, if not most, younger women in industrialised countries are at least moderately dissatisfied with their body weight or shape [42]. The fact that body dissatisfaction is "normative" does not imply that it is harmless; instead, it should be given more attention as a public health issue (Mond et al., 2013). In line with this, most young adult women reported constantly struggling with the number on the scale and whether they could fit into an ideal size.

*“Looking good is the top motivator.”* [43]

*“I have always wanted to become slim, and I still do.”* [44, 45]

*“The scale is probably the biggest motivator.”* [43]

In many cases, the eagerness to lose weight and change body image proceeds the willingness to be healthy; initially, it is a powerful drive. Rather than see PA as a good thing, these women see it as a way to achieve the ideal body shape and weight [46]. Particularly when some women do not see health as an issue since they are still young, they would devote more effort to reaching an ideal image.

*“I really don’t care. If you don’t do anything, what good does endurance do me? I mean really! [laughter].”* [43]

Weight management and body image change typically from a general dissatisfaction toward women’s body; these facilitators often play a starter role for PA, yet, are not ideal for longtime adherence. Research has shown that body dissatisfaction is often posited as an extrinsic motivation (introjected regulation) and will usually backfire in time [47]. In line with this, young adult women also reported that health benefits and achievement is tempered by the lack of change in their appearance or body image.

*“It’s great to have the strength and the cardiovascular ability and everything else but you still, you still look funny in your clothes.”* [48]

The constant dissatisfaction with body image and weight often turns facilitators into barriers which come with pressure, anxiety, embarrassment, lower self-esteem, etc. As negative feelings deepen over time, it is possible to cause further impairment in mental health and physical health, such as body image disorders and eating disorders [49] and compulsive exercise [50]. These consequences are not rare, and it violates one of the fundamental goals of PA, which is health.

##### Weight stigma & social embarrassment

As PA can be an individual activity, very often, the contexts in which it takes place and some activities are social. Apart from the self-perception of body dissatisfaction and weight, the pressured and sensitive mindset leads to social comparisons and feelings of body-focused self-consciousness around others.

*“I almost felt embarrassed...when you’re standing in the dressing room and you know that you have gained weight and you’re not comfortable....”* [44, 45]

Young adult women, particularly overweight women, reported often experiencing weight stigma in a social-based PA environment. They described feeling uncomfortable and humiliated as a barrier to PA. The lower self-esteem and fear of physique judgments from others keep bringing them a sense of failure and embarrassment. This circumstance existed not only in a PA environment but also in daily contexts. Namely, overweight women reported very often being subject to name-calling from peers in social interactions, with terms such as *sister boom* or *fatty boom boom* [51].

“*There is, like, social things that come with being fat. There is the name-calling.”* [51]

Beyond the self-perceived embarrassment, overweight women were frequently advised by others (family, friends, media, etc.) that if they simply moved more, they would lose weight and be healthier [52]. Under the same logic, PA programs frequently adopted a weight-centric approach, to engage beings to acquire a socially desired body size and, eventually, health. The terms "weight" and "health" are sometimes used interchangeably, which may contribute to PA-related weight stigma [52]. Namely, this group reported disliking the use of descriptor words with negative connotations, such as “obesity” and “weight loss”; instead, words like “fitness”, “health”, and “well-being” are more acceptable when doing PA as they do not engender weight stigma connotations [53].

*“… And I sort of looked at the wording again and I felt like—it was just really professional and it felt like it was respectful, so I felt pretty confident that it would be a positive experience...”* [53]

When society promotes a healthy weight and unattainable “ideal” body image, it is easier to understand why overweight or obese are stigmatised and denigrated. Epidemiological research has also shown that both the risks of obesity and the advantages of weight loss can be exaggerated. The obesity epidemic rhetoric is fueled partly by political, economic, and socio-cultural forces [54]. It is argued that this discourse is fuelled by a toxic mix of equivocal scientific information about the causes and consequences of obesity, as well as moral and ideological agendas primarily based on the rhetoric of decline in Western attitudes to diet and PA [55]. The impact of weight stigma on women’s PA is complicated and contradictory; it motivates young adult women’s PA yet elicits various unpleasant emotions, such as humiliation and shame.

##### Body image & beauty standards

With most young adult women reporting body dissatisfaction and weight stigma as both facilitators and barriers, some of them also criticised the unattainable contemporary body image and the single beauty standard that kept pressuring them from the societal and cultural level, namely overvalued thinness.

*“I don’t think they [the media] say anything about the amount you should be exercising, just that you should be thin.”* [56]

From the media and “others”, no matter for overweight or normal-weight women, thinness is prescribed as equal to health. As aforementioned, many programs also closely link weight to health, which presents a fundamental thought that exercise makes people thinner and that getting thinner is the same as becoming healthier. Yet, only emphasising health seems not as powerful enough for young adult women. Beyond health, there is a further underlying thought which is thinness equal to beauty, equal to self-disciplined success; however, this often falls into a single standard, which is the “Western White standard”. For instance, Non-White women have spoken about feeling under pressure from "White standards of beauty" and cultural conflicts over body image. Black women allegedly felt "bitter" over conforming to the White cultural ideal of beauty.

*“This is a country that values appearances*… *Jennifer Lopez, she looks like an American now. Yeah and she’s lost her uniqueness.”* [57]

*“I walk into the gym, and they all look like Workout Barbie— blonde-haired, ultra-slim, big chest, super-thin thighs. Only I don’t look like Workout Barbie. If anything, I want to look like Halle Berry. She’s got a nice little shape, she’s got hips.”* [57]

The “Western White standard” could be explained by the ideology of advanced capitalist societies that are reproduced at the site of the body through the mode of working toward bodily perfection. This ideology has encouraged critical evaluation of women's physical attributes, fostering competition and envy among women and encouraging the pursuit of impossible-to-achieve/to-maintain goals [58]. Namely, women posit that being an overweight man was often regarded as a sign of success (not in all situations), whereas being a perfectly shaped woman was just considered one aspect of success.

*“Because I want to be a lawyer, she’s like ‘How are you going to walk around the courtroom in like (that body)?’…… ‘Ooh, but you can’t be fat on the news. You gotta be skinny. You know what I mean?’”* [57]

In fact, the contemporary critique of sociocultural constructions of body aesthetics from many disciplinary perspectives has long existed [59–62]. It is argued that the body no longer serves only as an individual's entity but as a mirror of society's culture and ideology. As globalisation advances and people from diverse cultural identities coexist in the same social milieu, the dominant culture (Western-White) pressures minority cultures and is reflected in women's body image. Furthermore, women’s narratives often suggest PA is “fitness,” “gym”, and “working out.” Only a few discuss PA in connection to sports (“being athletic,” “doing sports”). This key distinction indicates that young adult women have appropriated a dominant discourse about sport and PA, a gendered discourse that holds that sport is for men more than women and that exercise or specific body modification toward PA can be ideally marketed to women when it rearticulates a conventional discourse of femininity [46]. Under this discourse, young adult women equate PA with self-confidence, self-discipline and holistic well-being. They position themselves as a subject within mainstream bodily discourses and construct themselves as subjects who negatively relate to the self (desire a thinner, more beautiful body closer to the Western ideal) [46]. Women's choice to PA is limited by these underlying social expectations on gender, which advocate for women's liberty to pursue their bodies while simultaneously reinforcing and outlining body standards. Young adult women believe they are making greater choices when they often fall into the “beauty and health” trap society has built for them during this emotional paradox.

#### Interpersonal & organisational level: multiple roles, social support, and PA

##### Multiple roles & patriarchal family

Young adult women’s multiple roles are a critical trait that may stop them from engaging in PA. To begin with, the fact that women bear many duties in multiple roles does not negate the fact that men also confront multiple roles simultaneously. What is discussed here is when women are under more pressure from society when both genders face the same situation. Young adult women are themselves employees, wives and mothers. Multiple roles can be advantageous or problematic to one's overall well-being, depending on one's subjective perception of role management abilities [63, 64]. In studies, the role balance between the mother and herself is most acute. Family commitment and lack of time are the two most significant barriers at the interpersonal and organisational levels. Although women also play multiple roles in middle-to-old age, the duties of each role fade over time, namely, less domestic and educational responsibility, their job becomes more stable or retirement, etc.

In a typical family setting in current society, the nuclear family is considered the basic unit of the social order within a patriarchal discourse of the family, meeting emotional and practical needs in different ways depending on whether one is a man or a woman [65]. Men with a father identity are considered "naturally" suited to be heads of the household. Their significant obligations are tied to attributes such as fortitude and providing material requirements (e.g., food and shelter). When these attributes are fulfilled, they are “encouraged” to seek fulfilment outside of it (e.g., leisure pursuits). Women, the mother identity, are considered “naturally” suited to a domestic role, with their primary responsibility of household labour and childcare because they are positioned as nurturing, patient and understanding. However, it is difficult to measure the fulfilment of women’s family responsibility, so they are often less encouraged to pursue other fulfillments (e.g., exercise, personal hobbies) and identities (e.g., career woman, exerciser, athlete) outside of the home, because they are “fulfilled” through being good mothers [66, 67]. In this mentality, when women participate in PA for personal purposes, they not only receive societal judgment but would also feel guilty about their families and children and further believe themselves as bad mothers or not qualified mothers.

*“I think that’ll be a difficulty, to not be feeling guilty like ‘now I’m gonna go off and work out without my kids.’”* [67]

Women’s sacrifice is appreciated in the patriarchal society's and family's ethical systems. Nevertheless, it is worth reflecting that though most women regard family responsibilities as a significant barrier to PA, they do not consider that they should change their current priorities. Despite women's frustrations with the sacrifices they made for their families, lack of “me” time, lack of help from others, and “doing it all” were eventually accepted as a normal part of their life and also social norms [67–71].

*“I’ve started talking with some of my girlfriends that men do that; they just sit back and relax after work and don’t help out! It’s just the way they are.”* [67]

*“In our culture men usually do nothing at home. Women do the cooking and the cleaning, even if they work. If they want to do something, they don’t have the chance.”* [72]

With this ongoing lifestyle, many women state that the home chores take the place of their PA, which can be seen as domestic PA.

*“I think ladies, they feel like they are doing household work so it is exercise, and they do not need to do anything extra.”* [73]

##### Motherhood & children’s needs

In terms of the patriarchal social norms of women’s family responsibilities. In truth, the children's expectations also play a vital role. In simple family routines, when children need to eat (often given by their mothers) or participate in activities that necessitate their mothers' company, women frequently re-prioritize their demands and put their children's needs first. PA, a leisure demand rather than a requirement (as opposed to a job, etc.), becomes a sacrifice of the child's needs at such times and is often the first to be pushed aside. Social norms should not be the only condition, part of the pressure in this competition between the priority of the mother and children comes from the child's expectations or demands (external pressure), and part of it comes from the mother's sense of obligation to the child (internal pressure), which is motherhood.

By not emphasising and discussing the political and cultural definition of motherhood, but only the part of women's nature [65, 74], women often prioritise their children before them [75, 76]. It is not difficult to understand some women willing to give up their own leisure in this context. Young adult women, particularly first-time mothers, have to devote more time and energy to caring for their children, who rely heavily on their mothers. If the child's requirements and the mother's needs are combined, the issue is solved. Many women also note that if the child needs PA, or PA would help her better support the child and family, it is also a feasible option.

*“I go to the park daily. My kids insist that ‘Mamma, let’s go to the park’.”* [73]

*“I noticed when I exercise, I have more energy for them.”* [67]

*“We enjoy being together and doing physical activities and that’s a good point for me and for them [children] and also for the relationship between mom and children.”* [77]

Family commitment is one of the most significant barriers to the multiple-role situation for young adult women. On the other hand, they perceived any support, especially family support, as a strong facilitator.

*“Some women don’t have help - the children have dads that don’t help or other family members, so that’s why too. They would have to put the children first. But I have help, luckily. That’s why I decided to start doing something [going to the gym].”* [69]

This helps to explain why young adult women indicate that family support is a crucial facilitator in their PA. When the stress of domestic tasks and caring can be shared with others, time becomes less of an issue, and they may devote more time to themselves. Time restrictions and competing priorities, such as employment, childcare, and household chores, always exist in women's lives. However, picking the priority requires a lot more effort, which cannot be accomplished only by women but by everyone around them.

#### Intrapersonal and community-level: religious identity, cultural identity, and PA

##### Muslim identity and PA

Most studies referred to religious influence in this review on Islam Muslims from various socioeconomic origins, languages, and races. Muslim people commit to their shared faith and continually strive to maintain their identity in non-Islamic, Muslim minority contexts [78]. At the same time, the argument and research towards Muslim women’s PA have been a popular research topic.

To begin, it's worth noting that while Islam does not prohibit women or its followers from participating in any PA, there are only specific requirements for dressing and socialising that may restrict Muslim women's PA more than other non-religious or non-Islamic women [46, 77–79]. From an outsider's view, Muslim women's strict religious rules are often perceived as being somewhat restrictive or conflicting, such as the most well-known rules: dress code and sex segregation. But Muslim women do not hold a contradictory view of the doctrine and their behaviours. Most women believe that their religious stance trumps everything else.

*“[Islam] influences almost all [of my everyday decisions]. I always consider what I am doing. Will what I will be doing be Islamicly wrong, or is that okay? …… The Quran, the Holy book…it consists of rules on how you should live your life and you should obey them.”* [79]

They also state that Islamic culture is dynamic so that they can embody complex identities regarding their socialisation, religiosity, and ethnicity. From the belief aspect, Islam is considered a facilitator which encourages Muslim women to be more active.

*“I don’t think it is anything to do with religion because it is all about the people and how they want to live their life”, and wearing a hijab will not affect the practice of PA.”* [77]

These women first identify themselves as Muslims. Within this devotion, if they do not obey the rules to participate in PA conveniently, it will be against their religion and threaten their piety. Notably, this does not imply that Muslim women are averse to PA. They share a positive attitude toward PA, seeing it as a way to improve their health, shed weight and shape, and socialise just like others. Only this attitude is founded on religion taking precedence over PA. This was defined as a "modern religious attitude" that usually lies in young Muslim women brought up in the West [80].

Therefore, the perceived barriers to being more physically active for these devout Muslim women are not rigid adherence to the doctrines but their limited alternatives and places for participation. Studies commonly reported that religious-related barriers are poor opportunities provided for women-only sites in gyms or sports venues, which violates the rule of not mixing with men in Islam. Also, only a few places accept the dress code, such as wearing a Hijab. The mismatch between Western and Islamic cultural traditions is a problem for many Muslim women, especially regarding privacy and their ideas of body modesty. The lack of culturally sensitive PA-related environments for Muslim women indicates they often experience a marginalisation of their recreation needs, and most PA places and programs are designed to cater to the “general” population. This is undeniably a position that ethnic and cultural minorities frequently encounter in society, not just in PA but also in physical education and other social levels. However, when mainstream and cultural outsiders only see the barriers as their strict requirements, it places the idea that minorities are expected to conform to the norms of the dominant society [81], which this idea and attitude may not be an acceptable and preferred way to Muslim women to volunteer in PA.

##### Embodied cultures

From the seeming conflict between religious values and the “main” PA promotion, it is worth considering the PA cultural differences. A researcher outside of other cultures is likely, to begin with the notion that PA is significantly vital, regardless of the cultural aspect or health aspect, and that everyone should and wants to engage in it. However, in diverse cultural contexts, it is necessary first to identify the role and credit of the PA behaviour itself.

A common thread across different culture groups was that PA is part of health promotion along with proper nutrition, caring for people’s mental well-being, physical health, and socialising. However, considering the high or low value given to PA by different cultures, the idea of encouraging women to engage in PA, or more precisely, activities that exercise and shape the body, is more Western in origin. Putting the health and gender equality judgment aside, some women state that leisure-time PA was not “normative” in their cultures, such as women who agreed that PA was not prioritised in South Asia culture, especially for girls and women.

*“Yeah, especially the older people like the in-laws. They say, ‘you don’t have to go to the gym. You do at home. We never went to the gym.’”* [73]

*“Even in the modern family, even in my family, I have seen if a girl is from a good family and she goes to the gym as well as does her study and all. But after marriage, she is doing nothing.”* [73]

Further, women reported the idea that women should be physically active and fit was also intensively conveyed through Western media.

*“My dad watches Indian channels, which is where I could see it, but as I say, those channels come from India and they don’t really push health as much as we do here.”* [56]

Women from Pakistani culture also remarked that PA is frowned upon by their communities and relatives. Further, sports are not seen as promoting ideal femininity within Pakistani society.

*“I remember I was bullied by the Pakistani boys because they thought I was too Norwegian. I played football. . . . The boys didn’t think I behaved like a Pakistani girl. . . I have got negative comments from people I barely know. They said it isn’t good for girls to exercise a lot.”* [82]

Families tend to urge young women toward "feminine" activities that are perceived as upholding traditional culture's notions of modesty and conventional femininity. [83]. Women who challenged traditional gender roles faced disapproval or harassment, with consequences ranging from becoming the subject of rumours or being labelled "too Western" [84]. Although certain cultural traditions impose constraints on women's behaviour, what should not be dismissed entirely is that PA can be merely a way in which these women subvert stereotypes emphasising the passivity, docility, and uncleanliness of women of South-Asian descent. But for women who give less appreciation and priority to it, it may be just a part that can be discarded.

*“We can go swimming at school if we want to, but I haven’t. I don’t know if it is because I have become shyer. I don’t feel comfortable in a swimsuit…I had the opportunity, but I couldn’t cope. Not because my parents told me not to, but because I didn’t feel comfortable.”* [82]

Further, in South-Asia cultures, academic achievement was more critical than PA, from parents' and young adult women’s views.

*“You’re going to get more in life by studying than going to the gym or playing sports, so (laughs). That’s the way it was when I was growing up to be honest.”* [56]

*“There is sports, but us as we grow up, we say we don’t focus to those things…”* [51]

*“When I go to the gym, I try to bring my study material and try to study on the elliptical. If I don’t have study material, I feel you know, I am wasting time when I could actually work for the test.”* [73]

In the examples, culture is experienced as a restriction on young adult women’s PA lives since culture and religiosity are perceived as practices that the girls learned as young children, which are often taken for granted. Nevertheless, when different cultures are encountered, they can be points of departure for hybrid and reflexive practices.

*“When my sister and I first started playing soccer here, my father would never come to watch us play. He really didn’t approve. But once he saw that everyone else does it here and that we are good—now he comes to all the games. It would have been different if he had a son.”* [57]

Quite different from the abovementioned religious barriers, some women also pointed out that religious community leaders are not giving much attention to PA.

*“So, I think that, sometimes, culture goes over religion and overshadows it.”* [46]

From both perspectives, it is evident that religion and culture are overpowering but flexible and embodied in women’s PA practice. Women’s bodies can both be inscribed with vehicles of culture [85]. These gender-based racial and ethnic disparities in PA are rooted in their cultures, communities and families, which start in childhood, suggesting that culture-specific beliefs about PA's role in women's lives have profound influences on lifelong PA. Yet if merely from a cultural standpoint, it is worthwhile to investigate what values PA stands for in different cultures. Designing culturally based programs for women might also be more beneficial.

#### Environmental & policy level: safety issues and women’s fears

*“No, I don’t feel safe because we have drug addicts, traffic, women trafficking it’s not safe for us to walk in the streets.”* [51]

Women’s safety concerns about the environment are the most outstanding barriers reported at this level. Safety concerns are usually described as fear of personal safety when walking or cycling in the dark or fear of being harassed [86]. Many women emphasised feeling unsafe being outdoors alone.

*“Where I live, you’re “frait long man” (scared of the man); it is not safe anymore. You might be raped or something.”* [56]

The gendered safety concern is one of the primary reasons. Sexual harassment and other forms of sexual violence in public spaces are everyday occurrences for women and girls worldwide, leading them to experience stress and fear in settings of everyday life (Ceccato & Loukaitou-Sideris, 2022).

*“I know for a fact that I can’t walk with a miniskirt in Jo’burg. Because [. . .] it’s insults continuously. Because you have men trying to follow you, men trying to ask you this, men trying to say that ...”* [51]

The fear of sexual victimisation may prevent women to participate in PA in school, work, and public life, limiting their opportunities [87, 88]. Women believe that men's violence is distributed unevenly across places and times. They learn to recognise the danger of unexpected individuals in public places [89]. This self-protective awareness and gendered fears are natural and raised early.

*“Compared to my male cousins, when I was growing up–there are like one or two who are older than me–I think they were allowed to like, go on the road and ride the bike and stuff like that, but we weren’t allowed to go and I knew that was because I was a girl so I think, but I didn’t stop me from–I don’t know how to describe it but I didn’t feel it was a bad thing that they were not letting us go cos I just thought like because they wanted me to be safe.”* [56]

In line with this, women reported that the potential to do outdoor activities or go out is raised when the environment is safer.

*“Safety is higher than in KSA and freedom is more so there is a lot of potential for walking.”* [77]

## Discussion

This qualitative review offered in-depth and holistic evidence of the young adult women's self-identified barriers and facilitators, and there was a relatively even distribution of factors across the five levels of SEM and between barriers and facilitators. The qualitative synthesis showed the contexts of barriers and facilitators and intertwined circumstances between different levels.

In this review, the term PA was used to identify studies without distinguishing its different domains. Still, each of the four domains of PA is covered in the result: the occupational PA is reflected at the organisational level; the transportation PA is reflected at the environmental level; the domestic PA is reflected at the interpersonal level and the organisational level, respectively; and leisure-time PA crosses all barriers and facilitators. In fact, it was noted that although most of the included studies used the term "PA", the participant narratives focus on leisure-time PA in general. The included study also found that PA is frequently perceived as a form of leisure activity rather than any physical movement that can be accumulated across various activities [90]. Young adult women in this review often referred to PA as planned, structured, and repetitive movements with a set schedule and duration, which fall within the definition of exercise [91]. Some women also mentioned sports, but “exercise” or “workout” in the gym was more frequently reported. It seems young adult women are prone to amplify leisure-time PA above the other three domains when considering their PA. This may be connected to their attitudes and motives since young adult women perceived having fun, learning new skills, managing weight, improving body image, socialising, relaxing, etc., as facilitators and goals, which typically link to leisure-time PA. Furthermore, being healthy and young may potentially reduce engagement in the other three PA domains, as staying healthy is not the strongest or most urgent facilitator. At the same time, given women's negative attitudes toward domestic activity, such as housework, they may view PA other than the leisure-time domain as an additional burden. Therefore, future strategies towards leisure time PA may be more appealing to young adult women; occupational, transportation and domestic PA may be considered extra-load or less appealing. However, these three domains are also essential for those who genuinely do not have a scheduled time for PA.

Similarities of barriers and facilitators to PA shared by young adult women or even further shared with middle to older women from previous research were frequently culturally and gender-based. Health, PA knowledge and skills, body image, multiple roles, social support, societal and cultural influences, and safety issues were some outstanding aspects. The differences in the prevalence between barriers and facilitators were not precisely divided by age but by the occurrence of life events and adaptation of social roles. One reason could be that only a few studies explicitly reported participants' ages, and only a few women emphasised age in narratives. Alternatively, women preferred to describe these factors with respect to their life stage or events but not with regard to their biological age. Young adult women between the ages of 18 and 40 typically experience four key life events and transitions: transition into higher education, transition to employment, transition to marriage and other committed relationships, and transition to motherhood (pregnancy/having a child)[92]. Changes in roles frequently entail changes in responsibilities and way of life, such as becoming a self-sufficient (financially) adult, juggling work and self, becoming a wife or mother, juggling work, family, and self, and having great obligations with family. It is worth noting that these social roles are overlapped rather than altered when women progress through different life events and transitions. This explains why "lack of time" was recognised by nearly all young adult women as the primary barrier to PA. Due to the overlapping roles and commitments (such as tiredness from juggling excessive workloads, household chores, and family obligations), young adult women find it challenging to carve out time for themselves, let alone for PA. This result was consistent with earlier research showing that most young adult women experienced a significant personal change in their 20 s [93], beginning work, changing from being single to cohabiting, getting married, and getting pregnant/having a child all decreased their PA [92]. It also highlighted that young adult women often faced more social-cultural barriers to PA at the interpersonal, organisational and community levels.

Beyond the differences between 18 to 40 years of women, the barriers and facilitators faced by young adult women differed from middle-aged and older women. One of the notable differences was the attitude toward health. Although women across ages generally reported health as the fundamental facilitator and motivation of PA participation, the perspective and priority on health seemed to differ. Previous studies found that health is the most often cited barrier and facilitator for PA by mid to old-age women [94]. For middle to old-age women, health is a significant and prioritised facilitator to PA, such as maintaining health status, preventing diseases, or as an approach for rehabilitation. Health conditions such as physical limitation and fear of injury are also considered barriers to more vigorous PA. Young adult women, on the other hand, did not see health as the strongest facilitator and rarely reported health conditions as a barrier; instead, social-cultural reasons were perceived to be the main influences. One reason could be that people do not pay too much attention to health when they are in good status since the population in this review is healthy. In addition, another explanation could be that young adult woman is strongly affected by and intensively interact with societal and cultural contexts, which stimulates and cultivate acquire autonomy and keep building a sense of self [95]. Hence, they sense and realise social-cultural influences fall on their life heavily, which includes their choice and engagement in PA.

Different from older women, a considerable amount of young adult women stated that the body change goals such as weight control (mostly losing weight), getting in better shape, etc., at the intrapersonal level was the strongest motivation and facilitators for PA. This finding is consistent with mounting evidence that suggests that body dissatisfaction is normative and relatively stable across women’s lifespans [96, 97]. The previous review found that body dissatisfaction was remarkably stable across the adult life span for women, at least until they were quite elderly, while the importance of body shape, weight and appearance decreased as women aged [96]. This may explain why body change was a strong facilitator, especially for young adult women. Additionally, social interaction, social culture, and health are all connected to women's irrational desire to alter their body image. For instance, the goal of body change for overweight women was frequently linked to health and social embarrassment of the body, while women with average weight also emphasised body image. More women today aspire to have the ideal body that conforms to cultural and social expectations of beauty and success in the West [45, 97, 98]. Young adult women are inexorably caught up in the chase of the perfect body, despite that some women also pointed out that this notion may not be realistic. It follows that, on the intrapersonal level, body image-oriented PA programs may be more attractive to young adult women. However, participation aimed at body transformation may not be long-lasting. It may come with adverse effects, so there is still a need for a more in-depth and holistic exploration of the relationship between young women regarding body beauty and health and exercise to find a balanced way to promote young adult women's participation in PA.

Research at the end of the last century suggested that one of the most challenging tasks for women was breaking gender stereotypes [1]. However, women are struggling with gender stereotypes and cultural norms regarding their roles and beauty after two decades. When a woman is mentioned, she is referred to first as a woman and then as a person. A man, on the other hand, is often regarded as a person first and a man second. Women face more environmental, cultural, and social barriers to PA than men. These challenges cannot be conquered only by women's resolution but by a joint effort. Most existing interventions continue to be situated on changes at the intrapersonal level. However, early research pointed out that proponents of individually oriented lifestyle behaviour change strategies were accused of supporting a victim-blaming ideology that serves as justification for retrenching rights and entitlements [36]. Most environmental toxins are dismissed with a wave of the hand, and it ignores the vital link between individual behaviour and social norms and rewards [35]. Thus, SEM was an appropriate tool to understand the juxtaposition between the individual and society and the subtle repercussions.

Young adult women’s barriers and facilitators to PA are complex and intertwined. Based on continuous education on personal behaviour change, what needs are progressive, large-scale, multilevel PA promotions in every dimension. Others and women must self-appreciate rather than exercise self-punitively to achieve an impossibly fashion-statement physique. Young adult women should first embrace PA, believing girls and women of all shapes and sizes deserve it [1] and that PA is beyond merely a means to achieve the ideal body and the consequent admiration. Since the environment and policies have a broad and profound impact on people, national policymakers should work closely with regional governments to support women's initiatives and opportunities for PA at a higher level. At the same time, it's important to note that increasing women's participation in PA won't happen overnight but rather via their continued commitment. Long-term tracking and gentled behaviour-changing approaches may have a more enduring influence than the efficient short-term program.

## Strengths, limitations and recommendations

This review employed a widely applied framework, the SEM, to classify barriers and facilitators. Participants' narratives were then used to synthesise the interactions between various levels of outstanding barriers and facilitators. By doing so, this review highlighted barriers and facilitators of PA participation with contexts rather than only as isolated components. The synthesis shows that barriers and facilitators are complex and convertible rather than entirely distinct, revealing the complexity of real-life PA participation and people's attitude toward it. These findings can compensate for the gaps in quantitative research and reviews by providing in-context information. Generally, this review offers vital empirical and practical insights for research and PA interventions for young adult women.

This review has several limitations. The participants' sociodemographic information (including the area of residence, occupation, education level, and income) and PA level was extracted to be analysed during the data extraction process. Yet, the authors were unable to compare the participants' sociodemographics with their reported barriers and facilitators, such as urban and rural women, high-income and low-income women, etc., in a systematic way because only a small number of studies provided detailed and complete sociodemographics, many articles only provided partial or no such data. Moreover, reviewers could not distinguish precisely between the various PA levels and barriers and facilitators because so few publications described participants' daily PA levels. When applying PA strategies and interventions, the components are also crucial; in-depth research on these factors may be done in the future.

## Conclusion

This qualitative synthesis revealed in-depth information on barriers and facilitators influencing young adult women’s PA. It highlights that the challenges young adult women face are diverse at different levels yet holistic and intertwined. Future studies of young adult women's targeted PA promotion initiatives should consider the SEM model as a holistic framework. The design and evaluation of upcoming effective programs should also leverage this paradigm, and qualitative study should be taken into account. To improve young adult women’s PA, only targeting personal behaviour change is not enough; organisations, communities, and policymakers must take action to provide opportunities for women’s PA at a higher level and broader range.

## Supplementary Information

**Additional file 1.**

**Additional file 2.**

**Additional file 3.**

## Data Availability

All data generated and analysed during this study are included in this published article [and its supplementary files from Additional files 1, 2 and 3].

## Abbreviations

PA: Physical activity
SEM: Social-ecological model
CASP: Critical Appraisal Skills Programme
WHO: World Health Organization
SDT: Self-determination theory
PE: Physical education
