# Supplementary material for: Barriers and facilitators to physical activity for young adult women: a systematic review and thematic synthesis of qualitative literature
Source: Int J Behav Nutr Phys Act. 2023 Feb 27;20:23. doi: 10.1186/s12966-023-01411-7 (PMC9972741; doi:10.1186/s12966-023-01411-7)
Supplement: Supplementary file 1 — Additional file 1. [file 12966_2023_1411_MOESM1_ESM.docx]

**Additional file 1**

**An example of the search strategy**

**Sport Discus:** Search modes - Boolean/Phrase

(physical activ* OR physical exercise* OR physical fitness OR sport* OR workout* OR physical movement OR physically active OR motor activ* OR active lifestyle*)

AND (adult women OR adult female* OR adult woman OR young women OR young female* OR young adult* )

AND (barrier* OR obstacle* OR challenge* OR difficult* OR issue* OR problem* OR limitation* or factor*)

AND (facilitator* OR enabler* OR motivation* OR influence* OR factor* OR determinant* OR feasibility OR opinion* OR thought* OR perception* OR perspective* OR view*)”
